# Harnessing the Potential of Artificial Intelligence in Supporting Older Adults

**DOI:** 10.1093/geroni/igaf122.1636

**Published:** 2025-12-31

**Authors:** Wendy Rogers

## Abstract

The landscape of aging and technology is changing dramatically. Uptake of technology is increasing among aging adults; technology is increasingly considered a solution to support needs of aging adults; and more technology products are marketed to older adults. Deployment of technology in healthcare and day-to-day activities is increasing and advances in technology such as artificial intelligence (AI) are increasingly aimed at supporting older adults. Yet, aging adults are often ignored in design, and robust research evaluating the usability, safety, and efficacy of these systems with older adults is limited. Even among older technology adopters, rapid changes in technology pose challenges in terms of the constant need for adaptation and continual learning. AI has the potential to provide support for older adults and enhance quality of life. We will present four projects aimed at harnessing this potential in myriad ways to provide support for older adults with and without cognitive impairment. Sara Czaja will focus on virtual reality as a method for supporting cognition, activities, social engagement, and technology proficiency. Walter Boot will share findings from evaluations of generative AI systems to provide decision support for older adults across a range of domains. Neil Charness will focus on the AI potential for the specific – and complex – task of Medicare decision making. Wendy Rogers will provide insights about the needs of older adults that can be supported by AI through an adaptive system. Together, this collection will provide valuable insights for AI to enhance everyday activities for older adults.

